# ClusCo: clustering and comparison of protein models

**DOI:** 10.1186/1471-2105-14-62

**Published:** 2013-02-22

**Authors:** Michal Jamroz, Andrzej Kolinski

**Affiliations:** 1 Laboratory of Theory of Biopolymers, Faculty of Chemistry, University of Warsaw, Pasteura 1, 02-093 Warsaw, Poland

## Abstract

**Background:**

The development, optimization and validation of protein modeling methods require efficient tools for structural comparison. Frequently, a large number of models need to be compared with the target native structure. The main reason for the development of Clusco software was to create a high-throughput tool for all-versus-all comparison, because calculating similarity matrix is the one of the bottlenecks in the protein modeling pipeline.

**Results:**

Clusco is fast and easy-to-use software for high-throughput comparison of protein models with different similarity measures (cRMSD, dRMSD, GDT_TS, TM-Score, MaxSub, Contact Map Overlap) and clustering of the comparison results with standard methods: K-means Clustering or Hierarchical Agglomerative Clustering.

**Conclusions:**

The application was highly optimized and written in C/C++, including the code for parallel execution on CPU and GPU, which resulted in a significant speedup over similar clustering and scoring computation programs.

## Background

The development, optimization and validation of protein modeling methods require efficient tools for structural comparison. Frequently, a large number of models need to be compared with the target native structure. There are numerous measures of model similarity. The most popular is the cRMSD – coordinate Root Mean-Square Deviation (after the best superimposition) [1]. The other popular scores are: GDT_TS – Global Distance Test Total Score [2], MaxSub – Maximal Substructure [3], TM-Score – Template-Modeling Score [4], or dRMSD – distance Root Mean-Square Deviation [5].

One of the methodologies most widely used for protein modeling includes performing the clustering step after generating a protein conformation ensemble [6-10] followed by the selection of a representative model (or models) for refinement. To achieve this, we need a similarity matrix of the whole ensemble, which contains all-versus-all comparison (for *N* conformers it gives *N*(*N*−1)/2 of score calculation). However, many available applications are not optimized for running time, because they were developed rather for simple pair comparison.

The main reason for the development of Clusco software was to create a high-throughput tool for all-versus-all comparison, because calculating similarity matrix is the one of the bottlenecks in the protein modeling pipeline.

## Implementation

The implementation of the similarity measures was performed using OpenMP API which supports multiprocessing programming. Additionally, the cRMSD algorithm was coded on the Graphic Processor Unit (GPU) architecture using nvidia cuda, which gave an over ten fold speedup in comparison with one CPU.

We used an open source parallel K-means [11] clustering code, implemented with OpenMP and serial C Clustering Library [12] for Hierarchical Agglomerative Clustering (single-linkage, maximum-linkage, average-linkage).

### Algorithms

**cRMSD** Coordinates Root Mean-Square deviation is defined as:

(1) $$\text{cRMSD}=\sqrt{\frac{1}{N}\overset{N}{\underset{i=1}{\sum }}∥x_{i}^{A}-x_{i}^{B}{∥}^{2}}$$

after the optimal superimposition. In this equation *N* is the number of atoms, $x_{i}^{A}$ - *i*-th atom position vector of protein *A*. There is no need to superimpose structures (calculating rotation matrix) to obtain the cRMSD. By the diagonalization of the 3×3 covariance matrix *M*, we obtain the cRMSD value by [13]:

(2) $$\text{cRMSD}=\sqrt{R_{A}^{2}+R_{B}^{2}-2(\sqrt{\lambda_{1}}+\sqrt{\lambda_{2}}+S\sqrt{\lambda_{3}})}$$

where *R*_*A*_ is the radius of gyration of protein *A*, *S* is the sign of the covariance matrix determinant, *λ* is the eigenvalue (sorted in descending order) of the square of the covariance matrix. These eigenvalues can be computed by finding roots of the cubic equation instead of computational demanding Singular Value Decomposition of the covariance matrix.

**GDT_TS** Global Distance Test Total Score is defined as:

(3) $$\begin{array}{ll} \text{GDT\_TS}= & \frac{1}{4}\left(\max C_{1\text{Å}}+\max C_{2\text{Å}}+\max C_{4\text{Å}}\right)\\ & \left(+\max C_{8\text{Å}}\right) \end{array}$$

where *C*_1Å_ is the number of atom pairs below the 1Å distance. Max denotes here the maximal value for a series of superimpositions.

The Global Distance Test algorithm is NP-hard [14], and all the GDT_TS computing algorithms use their own heuristics. Our GDT_TS algorithm is as follows: 1) divide the chain into all possible continuous 4,*N*/4,*N*/2,*N* fragments, and 2) use them as initial superimposition fragments (i.e. superimpose the whole structure by a rotation matrix computed by superimposing each fragment), 3) find atom pairs which are closer by cutoff (1,2,4,8[Å]), 4) select atoms which are closer than 3.5Å and use them for another superimposition until the number of selected atoms does not change in four iterations.

**TM-Score, MaxSub** (Template Modeling Score and Maximum Subset, respectively) [3,4] – both scores are variations of Levitt and Gerstein (1998) score.

TM-Score is defined as:

(4) $$\mathrm{TM}-\text{Score}=\max\left[\frac{1}{N}\overset{N}{\underset{i}{\sum }}\frac{1}{1+{\left(\frac{d_{i}}{d_{0}}\right)}^{2}}\right]$$

where $d_{0}=1.24\sqrt[{3}]{N-15}-1.8[\text{Å}]$, and MaxSub is defined as the TM-Score with *d*_0_=3.5Å. For the calculation of both scores we used the same searching algorithm as for GDT_TS, which means that the computational costs of GDT_TS, MaxSub, and the TM-Score will be the same.

**dRMSD** distance Root Mean-Square Deviation [5]. This score is the deviation of inter-molecular distance matrices:

(5) $$\text{dRMSD}=\sqrt{\frac{2}{N(N-1)}\overset{N-1}{\underset{i=1}{\sum }}\overset{N}{\underset{j=i+1}{\sum }}{\left(d_{\text{ij}}^{A}-d_{\text{ij}}^{B}\right)}^{2}}$$

where $d_{\text{ij}}^{A}$ is the distance between *i* and *j* atoms of protein model *A*. Note that the representation of molecule as a distance matrix causes loss of information about chirality.

**CMO** Contact Map Overlap [15]. Using a representation of the protein structure as a binary matrix *C*, defined as:

(6) $$C_{i,j}=\left\{\begin{array}{ll} 1 & \text{if}\;|x_{i}-x_{j}|<\text{cut-off}\\ 0 & \text{otherwise} \end{array}\right)$$

we use Sørensen Similarity Index as a similarity score between the two proteins *A*, *B*:

(7) $$S=100\frac{2n(A\cap B)}{n(A)+n(B)}$$

where *n*(*A*) is the number of contacts in protein *A*.

## Results and discussion

Using the cRMSD, dRMSD, MaxSub, GDT_TS, TM-Score, CMO as a similarity measure, Clusco can calculate all-versus-all (or with respect to the reference model) scores of proteins from a one-column list file or using multimodel pdb file. The calculated results may be used, for example, as a similarity matrix input for clustering algorithms or clustered by Clusco itself.

In this section we show the examples of usage and the performance of Clusco with respect to other similar programs. All tests were performed on a box with intel E5649 CPU (24 threads), nvidia GeForce GTX 470 GPU and 24GB of RAM. The computation time elapsed was assessed by the standard *NIX “time” program.

### Selecting of pairs of models within a given cRMSD threshold

Recently, Fogolari and coworkers [16] described an algorithm for reducing the computational cost of all-versus-all comparison of protein models using cRMSD by inverse triangle inequality. As an example of that idea, the authors provided fsss software and 1ctf protein models from 4state_reduced decoy set (Decoys ’R’ Us) [17] as an input ensemble. The fsss software outputs pairs of models with cRMSD below a given threshold (3.2 Å in this example).

We compared fsss and Clusco based on this dataset, recording the time spent by one CPU performing the task. Note that Clusco computes all-versus-all scores by default, and to get results similar to the ones obtained from fsss (only pairs below given threshold) we needed to filter the output by awk (standard *NIX program): clusco-l 4state_reduced_1ctf.list-s rmsd-o4state.tmp;awk‘{if($3<3.2)print$line}'4state.tmp > output.rmsd.

fsss software spent 149.14 seconds on this task, and Clusco+awk spent 0.46 (Clusco) + 0.11 (awk) seconds, which amounts to 261× speedup. Note that it is possible to improve these results, using parallel execution of Clusco (by simply defining shell variable OMP_NUM_THREADS before Clusco execution).

### Clustering of protein decoys from five independent molecular dynamics trajectories

The decoy set vhp_mcmd [18] from Decoys ’R’ Us database contains the results of five (NATIVE, F1, F3, F4, F7) Molecular Dynamics simulations of the thermostable subdomain from chicken villin headpiece (36 residues, pdb code: 1vii), starting from different protein conformations. The set contains 6256 of villin conformations in total, in the range of 0.49 - 12.8 Å cRMSD to the experimental structure deposited in the Protein DataBank.

Using cRMSD and each of the Clusco clustering schemes, it is possible to separate this decoy set roughly into former trajectories, as we show in Additional file 1: Table S1. Each of the Hierarchical Agglomerative methods divides decoys into rather separate clusters i.e. more than 85% of trajectory models create a separated cluster, while of “NATIVE” and “F1” models create one common cluster (which is the result of “F1” convergence to the native structure during simulation – we refer the interested readers to Figure two in [18]). Other clustering scheme in K-means results in the grouping of “NATIVE” and “F1” models into four separated clusters.

Command to perform clustering described above: clusco -l vhp_mcmd.list -s rmsd <0,1,2,3>8.

The running time for this dataset varied from 5 seconds (for K-means clustering and GPU cRMSD comparison), to 3.5 minutes (for average-linkage, Hierarchical Agglomerative Clustering and CPU cRMSD comparison, see Additional file 1: Table S1.

### Selecting representative model from *de-novo* modeling decoy set

Clustering of protein models after *de-novo* simulations is one the methods most commonly used for the selection of the representative model from the decoys set [6-10]. We compared Clusco with other clustering software (durandal[19], calibur[20], spicker[21]) in terms of results and computation time. To do this, we used public available I-Tasser [22] decoys set, containing 12500-32000 models for each of 56 modelled target protein.

calibur uses preprocessing of decoy set in three ways: screening-out unlikely candidates by setting lower and upper cRMSD bounds, using triangular inequality for assessing if particular model is within the threshold distance from a group of models (which reduces the number of structure comparisons), detecting and ignoring outlier decoys. durandal uses triangular inequality (like calibur) for the approximation of cRMSD value of randomly chosen decoy and fill-up distance matrix until it contains proper amount of information for the next, clustering step. spicker selects the decoy with the largest number of structurally similar decoys (by automatically detected threshold value) and creates the first cluster. The process is repeated to get a sufficient number of clusters.

Clusco was run with cRMSD as a similarity measure (just as durandal, calibur and spicker) and K-means as a clustering method. We set number of clusters to 20: clusco -l list -s rmsd 0 20.

The Clusco representative model was selected by min(<*R*>/*f*), where *f* denoted the fraction of elements in particular cluster and <*R*> – the average cRMSD between cluster elements.

For the comparison of software reliability, we calculated tm-score to the experimental structure (Additional file 1: Table S2) and Z-score of the tm-score (where Z-score <0 means that a model is worse than the average structure of the decoy set, for detailed results see Additional file 1: Table S3).

According to the average tm-score, all of the programs gave similar results: all, except durandal, gave the average tm-score 0.59, and durandal gave the score of 0.58. According to Z-score, the best algorithms were calibur and Clusco (49/56 of the models with Z-Score above zero), followed by spicker and durandal (45/56 and 41/56 respectively).

We recorded the execution time of each algorithm: durandal was the fastest (spending 140 minutes on the clustering of the whole dataset), then Clusco on one CPU (426 min), spicker (435 min) and calibur (856 min). If we allow for the possibility of parallel execution on GPU/CPU – Clusco finished calculations in 131 min on 4 CPU’s, in 106 min on 4 CPU’s and 1 GPU and in 47 min on 23 CPU’s. We summarized these results in Table 1.

**Table 1:** Total time for clustering of decoys

**Program**	**Total time(min)**	**1thx_**	**2reb_2**
spicker	435	10	4
durandal	140	9	0.9
calibur	859	64	1.2
Clusco 1CPU	426	32	1.8
Clusco 1CPU 1GPU	213	19	0.7
Clusco 2CPU	219	16	0.9
Clusco 2CPU 1GPU	146	12	0.4
Clusco 4CPU	131	11	0.5
Clusco 4CPU 1GPU	106	7	0.4
Clusco 23CPU	47	3	0.3

1thx_ – 32000 decoys of 108 aa. protein, 2reb_2 – 12500 decoys of 60 aa. protein. Need to note that spicker use maximum of 13500 decoys.

Analyzing the above results, we can conclude that Clusco gives results which are as good as the ones provided by the state-of-the-art calibur in half of the time, however, on the commonly used today multicore machines, our program gives results in the time about 18× shorter than calibur.

### Comparison of structures with reference/experimental model

To compute the score between multi-model pdb file (tra.pdb) and the reference structure (ref.pdb), one should run Clusco in the following way: 

This command will compute the tm-score for each of tra.pdb models, saving the results into output.txt. If OMP_NUM_THREADS variable was not set, program will utilize all available CPU’s.

We recorded the computation time of the tm-score to the reference (experimental) structure with Clusco and the original TM-Score software [4] using the decoy set mentioned in the previous paragraph. TM-Score performed the task in 68 minutes, Clusco on 1 CPU – in 53 minutes (speedup of about 1.2×), but when we ran Clusco on 12 CPU’s, it completed the task in 7 minutes (speedup about 10×) (detailed data in Additional file 1: Table S4).

It must be noted that the computation time for GDT_TS and MaxSub will be mostly identical, since all of these algorithms use the same method for selecting fragments of structure. Optionally, it is possible to compute more exact GDT_TS score with Clusco by using -s gdtExt flag – in this particular case Clusco will split structures into many more fragments.

**Figure 1:**
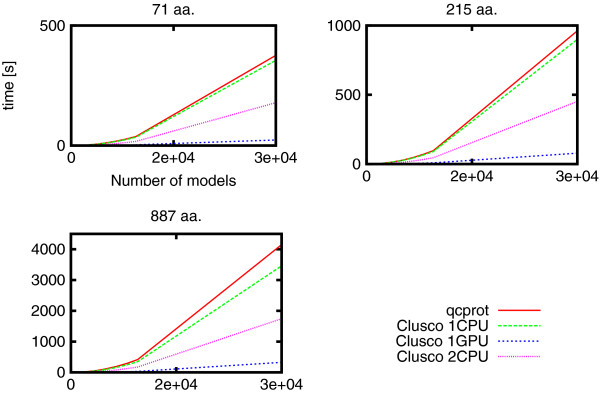
**Comparison of running time of all-versus-all Clusco and **qcprot. cRMSD computation for three proteins of different length (71, 215 and 887 residues). For *N* models it compute *N*(*N*−1)/2 cRMSD values.

For the comparison of cRMSD computation time, we used the qcprot algorithm [23] claimed by the authors to be probably the fastest available today. Recorded times were only for the cRMSD routine (without I/O time). In this comparison test, we got slightly better results than the qcprot: the speedup of 1.1−1.2× for Clusco on one CPU and the speedup of 12.7−16.1× on one GPU. See Figure 1 for details.

Recently Hung & Samudrala [24] published an algorithm for the computation of all-versus-all tm-score on amd GPU and CPU. We compared Clusco with this algorithm using the exemplary data attached to the program package (1000 models of 140 residues). Clusco on one CPU completed the computation in 53.65 minutes, Hung & Samudrala code – in 57.18 minutes, but Clusco can achieve pronounced speedup if executed in multi-CPU fashion (13.66 minutes on 4 CPU’s), which was not implemented in the Hung & Samudrala algorithm (see Figure S1 in Additional file 1 for tm-score values comparison). However, users with access to the amd GPU can complete this task significantly faster with Hung & Samudrala algorithm.

## Conclusions

We presented here versatile software for comparison and clustering of protein structures, optimized for novel multicore computers. We showed cuda implementation of cRMSD algorithm which may be usable for creating of proteins similarity matrices (a bottleneck of the clustering software) as an input for more efficient clustering algorithms.

However, up till now, not many computers are equipped with graphics cards capable of floating point computation, but most of them are equipped with multicore processors. Our software accounts for this situation by containing a parallel code for all described here comparison methods. It results in great-to-moderate speedup over an existing serial execution algorithms, together with clustering results as good as obtained using the state-of-the-art method, calibur.

Clusco is able to cluster small-to-moderate protein decoys with scoring functions other than the cRMSD, i.e. the TM-Score, dRMSD, GDT_TS, MaxSub, Contact Map Overlap, especially on many-core machines, which is unique.

Clusco may be useful for protein modeling community as an all-in-one, fast and easy in use software for daily lab work. It may be used as a standalone program for comparison or clustering of protein models or as a preprocessing tool for clustering algorithms, either as a compiled program or a fragment of Clusco’s source code.

## Availability and requirements

**Project name:** ClusCo**Project home page:**http://biocomp.chem.uw.edu.pl/clusco**Operating system:**gnu/Linux**Programming language:**c/c++, cuda**Other requirements:** OpenMP library (included in gcc ⩾ 4.2 compiler), optionally: cuda sdk and cuda compatible graphic card**License:**gnu gpl (scoring functions), Python License (Hierarchical Clustering library), custom license for K-means library (included in package)

## Competing interests

The authors declare that they have no competing interests.

## Authors’ contributions

MJ wrote algorithms, manual and manuscript. AK corrected the manuscript. Both authors read and approved the final manuscript.

## Supplementary Material

Additional file 1 **The Supporting Information.** Additional comparison results with other software. Click here for file
