# Supplementary material for: ClusCo: clustering and comparison of protein models
Source: BMC Bioinformatics. 2013 Feb 22;14:62. doi: 10.1186/1471-2105-14-62 (PMC3645956; doi:10.1186/1471-2105-14-62)
Supplement: Additional file 1 — The Supporting Information. Additional comparison results with other software. [file 1471-2105-14-62-S1.pdf]

Supporting Information
ClusCo: clustering and comparison of protein models

Michal Jamroz, Andrzej Kolinski
Laboratory of Theory of Biopolymers, Faculty of Chemistry,
University of Warsaw, Pasteura 1, 02-093 Warsaw, Poland

February 21, 2013

Clust. method	NATIVE	F1	F3	F4	F7
0	C1(48%), C4(52%)	C5(31%), C6(64%)	C3(13%), C0(87%)	C2(99%)	C7(98%)
1	C4(100%)	C4(98%)	C5(100%)	C7(100%)	C3(100%)
2	C2(100%)	C4(11%), C2(86%)	C0(13%), C5(87%)	C1(100%)	C3(97%)
3	C0(100%)	C0(95%)	C3(87%), C6(13%)	C4(96%)	C5(96%)

Table S1: Results of clustering by Clusco with cRMSD as similarity score and all available clustering methods. Number of clusters was set to 8. In parenthesis - percent of particular decoy models in that cluster. We show only clusters contains more than 10% of total number of considered decoy models. Clustering methods are: 0 - K-means Clustering; 1 - Hierarchical Agglomerative Clustering, pairwise, single-linkage; 2 - H. A. C., pairwise, maximum-linkage, 3 - H. A. C., pairwise, average-linkage.

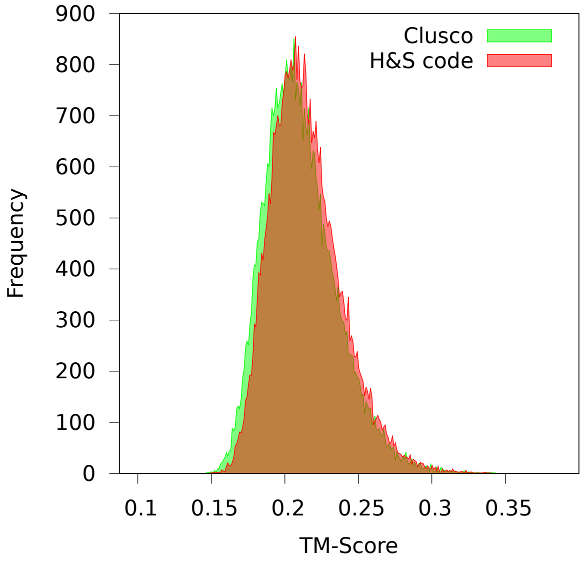

Figure S1: Histograms of Clusco and Hang & Samudrala code TM-Score values. Histograms generated from 44850 TM-Score values (comparison all-versus-all of 300 models). H&S code generate slightly higher values due to different implementation of TM-Score algorithm.

PDB ID	Clusco	SPICKER	DURANDAL	CALIBUR
1abv_	0.30	0.29	0.25	0.29
1af7__	0.50	0.46	0.46	0.49
1ah9_	0.58	0.51	0.48	0.65
1aoy_	0.69	0.66	0.66	0.66
1b4bA	0.44	0.41	0.41	0.48
1b72A	0.75	0.66	0.66	0.67
1bm8_	0.39	0.43	0.48	0.36
1bq9A	0.37	0.39	0.37	0.37
1cewI	0.74	0.72	0.72	0.73
1cqkA	0.85	0.85	0.85	0.85
1csp_	0.70	0.72	0.73	0.72
1cy5A	0.87	0.88	0.87	0.87
1dcjA_	0.36	0.35	0.36	0.37
1di2A_	0.80	0.80	0.80	0.77
1dtjA_	0.81	0.78	0.78	0.80
1egxA	0.77	0.77	0.77	0.77
1fadA	0.49	0.58	0.58	0.58
1fo5A	0.54	0.54	0.54	0.54
1g1cA	0.78	0.78	0.78	0.78
1gjxA	0.41	0.46	0.41	0.41
1gnuA	0.55	0.52	0.54	0.54
1gpt_	0.48	0.53	0.51	0.51
1gyvA	0.76	0.76	0.74	0.76
1hbkA	0.66	0.66	0.66	0.66
1itpA	0.32	0.34	0.30	0.31
1jnuA	0.75	0.73	0.73	0.75
1kjs_	0.36	0.38	0.38	0.38
1kviA	0.71	0.70	0.70	0.71
1mkyA3	0.41	0.41	0.41	0.41
1mla_2	0.62	0.62	0.62	0.63
1mn8A	0.35	0.36	0.30	0.36
1n0uA4	0.45	0.45	0.45	0.46
1ne3A	0.39	0.49	0.27	0.39
1no5A	0.42	0.42	0.42	0.43
1npsA	0.77	0.77	0.77	0.76
1o2fB_	0.39	0.36	0.44	0.36
1of9A	0.53	0.54	0.53	0.54
1ogwA_	0.86	0.86	0.85	0.85
1orgA	0.76	0.76	0.76	0.76
1pgx_	0.50	0.50	0.50	0.53
1r69_	0.72	0.75	0.75	0.75
1sfp_	0.72	0.75	0.75	0.75
1shfA	0.84	0.79	0.84	0.82
1sro_	0.65	0.65	0.65	0.65
1ten_	0.82	0.82	0.82	0.82
1tfi_	0.50	0.52	0.51	0.50
1thx_	0.80	0.80	0.80	0.80
1tif_	0.35	0.33	0.29	0.33
1tig_	0.54	0.42	0.50	0.56
1vcc_	0.39	0.40	0.37	0.38
256bA	0.76	0.76	0.76	0.76
2a0b_	0.78	0.80	0.78	0.77
2cr7A	0.44	0.48	0.34	0.39
2f3nA	0.73	0.73	0.73	0.73
2pcy_	0.64	0.64	0.64	0.64
2reb_2	0.33	0.32	0.34	0.34
Average	0.59	0.59	0.58	0.59

Table S2: Results of clustering of the *de-novo* predicted decoy set. Values are the tm-score of the representative structure. Clusco was run with cRMSD and K-means (K=20), other programs run with default parameters.

PDB ID	Clusco	SPICKER	DURANDAL	CALIBUR
1ten_	1.38	1.37	1.38	1.38
1o2fB_	0.57	0.03	1.49	0.03
2pcy_	0.50	0.70	0.50	0.75
1kjs_	-0.47	0.05	-0.05	0.05
1dtjA_	1.79	0.94	0.94	1.42
1mn8A	1.76	2.06	0.34	2.02
1gjxA	0.86	1.41	0.81	0.81
1ne3A	0.31	1.64	-1.19	0.37
1tif_	1.23	0.49	-0.72	0.74
1vcc_	0.62	0.83	0.34	0.38
1di2A_	1.18	1.20	1.18	0.59
1sro_	0.82	0.81	0.82	0.82
1mla_2	0.52	0.52	0.52	0.77
1thx_	0.62	0.62	0.62	0.48
1ogwA_	0.84	0.85	0.71	0.71
1bq9A	0.32	0.91	0.35	0.34
1orgA	0.37	0.38	0.37	0.37
1b72A	1.61	0.85	0.85	0.92
1abv_	0.17	0.11	-1.11	-0.03
1af7__	1.77	1.19	1.19	1.65
1g1cA	1.24	1.22	1.24	1.24
1bm8_	1.86	2.63	3.53	1.16
1tfi_	0.61	0.86	0.86	0.52
1aoy_	0.89	-0.19	-0.19	-0.19
1cy5A	0.66	1.09	0.67	0.67
1r69_	0.10	0.95	0.94	0.94
1npsA	0.86	0.80	0.86	0.55
1itpA	0.20	1.04	-0.42	-0.19
1b4bA	0.15	-0.56	-0.56	1.07
1tig_	1.12	-0.74	0.50	1.38
1gpt_	-0.44	1.07	0.28	0.46
2f3nA	0.87	0.86	0.87	0.87
2a0b_	0.47	0.82	0.47	0.24
1gyvA	0.68	0.48	-0.05	0.79
1csp_	0.25	0.95	0.96	0.76
1ah9_	0.79	-0.02	-0.36	1.42
2cr7A	0.12	0.64	-1.16	-0.52
1cqkA	1.52	1.48	1.48	1.52
1kviA	0.54	-0.01	-0.03	0.35
1fadA	-4.47	-0.01	-0.00	-0.00
1egxA	1.08	1.08	1.08	1.08
1dcjA_	0.23	-0.60	0.17	1.07
1n0uA4	-0.69	-0.68	-0.69	-0.50
2reb_2	-0.62	-0.74	-0.59	-0.56
1of9A	0.11	0.49	0.11	0.50
1jnuA	1.31	0.40	0.39	1.25
1fo5A	0.78	0.78	0.78	1.24
1pgx_	0.47	0.46	0.47	0.92
1gnuA	0.43	-0.48	0.11	0.12
1mkyA3	0.61	0.62	0.61	0.46
1hbkA	0.52	0.52	0.52	0.52
1no5A	-0.06	-0.06	-0.06	0.20
1cewI	1.21	0.51	0.50	1.02
1shfA	1.36	1.11	1.36	1.27
256bA	0.11	0.15	0.15	0.15
1sfp_	-0.22	1.68	1.63	1.63
%$Z > 0$	87.5	80.4	73.2	87.5

Table S3: Z-Score ($Z = (x - \mu)/\sigma$) of the tm-score for particular clustering result. Last row is the percent of cases (out of 56) where Z-Score was greater than 0 (better than decoy average).

PDB ID	TM-Score	Clusco 1	Clusco 12	Clusco 24
1abv_	1:14.20	0:52.91	0:06.72	0:05.89
1af7__	0:34.50	0:27.77	0:04.23	0:03.69
1ah9_	1:01.59	0:50.00	0:07.93	0:06.67
1aoy_	0:55.67	1:01.58	0:09.19	0:08.21
1b4bA	0:42.28	0:27.15	0:04.08	0:03.61
1b72A	0:15.49	0:14.44	0:02.55	0:02.39
1bm8_	2:00.33	1:16.00	0:09.73	0:08.66
1bq9A	0:31.88	0:24.41	0:04.14	0:03.68
1cewI	2:10.19	1:38.67	0:12.31	0:10.79
1cqkA	1:34.00	1:28.02	0:11.86	0:09.72
1csp_	0:25.03	0:25.97	0:04.07	0:03.32
1cy5A	1:48.14	1:59.06	0:15.86	0:13.35
1dcjA_	0:46.15	0:44.38	0:06.66	0:05.85
1di2A_	0:41.09	0:43.78	0:06.62	0:05.69
1dtjA_	0:42.66	0:49.38	0:07.44	0:06.38
1egxA	2:36.00	1:52.76	0:14.04	0:11.90
1fadA	1:37.10	0:46.09	0:06.28	0:05.28
1fo5A	1:57.34	1:02.54	0:08.32	0:07.36
1g1cA	1:45.20	1:22.22	0:11.66	0:09.12
1gjxA	0:41.91	0:31.89	0:04.44	0:03.99
1gnuA	2:25.26	1:36.84	0:12.27	0:09.99
1gpt_	0:40.02	0:35.06	0:06.32	0:05.38
1gyvA	1:22.38	1:07.36	0:08.43	0:07.24
1hbkA	1:40.80	1:08.13	0:08.97	0:08.18
1itpA	0:28.35	0:24.31	0:03.57	0:03.07
1jnuA	2:12.14	1:34.27	0:11.69	0:10.07
1kjs_	0:58.04	0:46.80	0:07.07	0:05.92
1kviA	0:36.66	0:42.75	0:06.25	0:05.66
1mkyA3	1:09.02	0:34.31	0:04.92	0:04.27
1mla_2	0:31.12	0:27.49	0:03.99	0:03.65
1mn8A	0:50.13	0:34.75	0:04.91	0:04.35
1n0uA4	0:39.60	0:27.88	0:04.11	0:03.34
1ne3A	0:22.95	0:18.00	0:03.18	0:02.53
1no5A	1:11.11	0:43.80	0:06.09	0:05.17
1npsA	1:16.98	1:08.52	0:09.15	0:07.77
1o2fB_	0:40.31	0:30.63	0:04.54	0:03.97
1of9A	1:31.64	0:52.40	0:07.51	0:06.42
1ogwA_	0:34.26	0:46.93	0:07.11	0:06.14
1orgA	2:36.38	1:58.51	0:15.14	0:12.32
1pgx_	0:45.71	0:32.48	0:05.37	0:04.63
1r69_	0:33.21	0:35.37	0:05.86	0:04.91
1sfp_	2:24.43	1:49.07	0:13.34	0:11.10
1shfA	0:35.21	0:32.99	0:05.12	0:04.73
1sro_	0:48.27	0:46.52	0:06.62	0:05.67
1ten_	1:12.48	1:07.55	0:09.13	0:07.73
1tfi_	0:39.18	0:34.35	0:06.21	0:05.61
1thx_	2:43.76	2:40.91	0:20.99	0:16.75
1tif_	0:19.47	0:19.30	0:03.23	0:02.84
1tig_	1:15.67	0:40.58	0:05.64	0:04.78
1vcc_	1:02.78	0:48.42	0:06.92	0:05.78
256bA	1:54.98	1:34.89	0:12.32	0:10.42
2a0b_	4:38.17	3:05.17	0:23.97	0:19.34
2cr7A	0:26.88	0:20.51	0:03.33	0:02.95
2f3nA	0:33.63	0:39.38	0:05.96	0:05.37
2pcy_	2:03.65	1:22.79	0:10.91	0:09.17
2reb_2	0:24.63	0:19.47	0:03.08	0:02.85
Total	68:10.01	53:7.51	7:21.35	6:15.62

Table S4: Running time of computing of the tm-score between decoy models and reference (experimental) structure. We show time of the original TM-Score algorithm and Clusco executed on 1, 12 and 24 threads.

1 Command-line options (clusco - -help)

```
USAGE:

   ./clusco_gpu  {-l <list filename>|-t <trajectory filename>} -s <rmsd
                 |rmsdGPU|drmsd|gdt|gdtExt|tmscore|maxsub|CMO|CMOn> [-d
                 <cutoff>] [-c] [-a] [-o <output filename>] [-e <reference
                 pdb filename>] [--] [--version] [-h] <clustering options
                 (only with -l flag)> ...


Where:

   -l <list filename>,  --list <list filename>
     (OR required)  List of files
         -- OR --
   -t <trajectory filename>,  --trajectory <trajectory filename>
     (OR required)  Multimodel file (PDB: MODEL .... ENDMDL)


   -s <rmsd|rmsdGPU|drmsd|gdt|gdtExt|tmscore|maxsub|CMO|CMOn>,  --score
      <rmsd|rmsdGPU|drmsd|gdt|gdtExt|tmscore|maxsub|CMO|CMOn>
     (required)  Select score for computation

   -d <cutoff>,  --cutoff <cutoff>
     Cutoff for Contact Map Overlap (default 3.8A)

   -c,  --cabstraf
     Multimodel TRAF (CABS) file format (default: PDB file format)

   -a,  --allatom
     Compute scores on all atoms (default: only C-alpha atoms)

   -o <output filename>,  --output <output filename>
     Output filename. Save results here.

   -e <reference pdb filename>,  --reference <reference pdb filename>
     Reference model (PDB file). Without this flag, program computes
     all-with-all

   --,  --ignore_rest
     Ignores the rest of the labeled arguments following this flag.

   --version
     Displays version information and exits.

   -h,  --help
     Displays usage information and exits.

   <clustering options (only with -l flag)>  (accepted multiple times)
     clustering options=

     K-Means: 0 OR 0 <K> (if only 0: K=3)

     Hierarchical, pairwise single-linkage: 1 OR 1 <K>

     Hierarchical, pairwise maximum-linkage: 2 OR 2 <K>
```

```
Hierarchical, pairwise average-linkage: 3 OR 3 <K>
```

```
Clustering and Comparison of Protein Models / GPL
```

2 Output files

For score-only computation, output looks like:

```
0        0      0
0        1    4.5
0        2   2.99
```

where first and second column is model index. For calculations to the reference structure, first column will be 0 in each row.

After clustering output looks like:

```
==> lista2.clustering0 <==
# Score: CMO
# Filename: lista2 (conformers: 1000)
# Hierarchical clustering, pairwise single-linkage, K=2

   0 :                 ./data/n_1.pdb :    0.65 : 1 : ./data/model1.pdb ./data/model4.pdb .....
```

where first column is the cluster index, second column - medoid structure, third column - average score within the cluster, fourth column - number of structures in this particular cluster

Another output looks like:

```
 ==> lista2.clustering1 <==
# Score: CMO
# Filename: lista2 (conformers: 1000)
# Hierarchical clustering, pairwise single-linkage, K=2

                ./data/n_1.pdb    1 *
                ./data/n_2.pdb    1
                ./data/n_3.pdb    1
                ./data/n_4.pdb    1
                ./data/n_5.pdb    1
                ./data/n_6.pdb    1
```

where first column is model path, second column is cluster for this model, third column (*) is cluster representant (medoid).
